# Technology of Petroleum Needle Coke Production in Processing of Decantoil with the Use of Polystyrene as a Polymeric Mesogen Additive

**DOI:** 10.1021/acsomega.1c02985

**Published:** 2021-07-23

**Authors:** Renat R. Gabdulkhakov, Viacheslav A. Rudko, Vladimir G. Povarov, Valery L. Ugolkov, Igor N. Pyagay, Ksenia I. Smyshlyaeva

**Affiliations:** † Saint Petersburg Mining University, St. Petersburg 199106, Russia; ‡ I. V. Grebenshchikov Institute of Silicate Chemistry of the Russian Academy of Sciences, St. Petersburg 199034, Russia

## Abstract

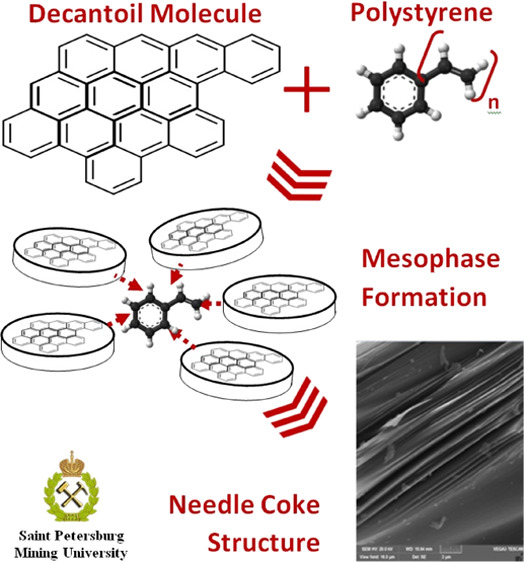

The results of experimental investigations on the coking of decanted heavy gasoil of catalytic cracking with polystyrene in a certain concentration range to obtain petroleum needle coke with the most developed string-base anisotropic structure and a microstructure point of at least 6.2 corresponding to the super-premium grade are presented. Certain regularities have been established to improve the structural quality index of the resulting needle coke from the optimal content of polystyrene in the base raw material, including the extreme dependence of the quality indices of needle coke on the polystyrene content (10 wt %). The decrease in the quality indices of the obtained carbon material is a consequence of uncontrolled changes toward an increase in the system viscosity performance (the viscosity increases 2.7 times). The experimentally obtained coefficient of thermal expansion (CTE) of needle coke-synthesized samples within the temperature range of 40–500 °C showed a reducing trend in CTE depending on the polymer additive proportion in the feedstock; for example, at 300 °C, the CTE decreases to 5.732 × 10^–6^ °C^–1^.

## 1 Introduction

Needle coke is a strategically important, highly structured carbon material obtained as a result of thermal degradation processing of highly fragranced petroleum feedstock.^1^ This type of carbon material has a developed fibrous structure,^2^ has a low coefficient of thermal expansion, high electrical conductivity, and low contents of heteroimpurities and sulfur,^3^ and is widely used in the electrode industry,^4,5^ in the production of lithium-ion batteries. Super-premium (SP) grades of this carbon material are used in the production of high-quality UHP (ultrahigh power) graphite electrodes for ultrahigh power electric arc furnaces.^6,7^

The quality of the resulting needle coke directly depends on the content and properties of the feed, namely, on the content of various groups of hydrocarbons,^8^ heteroatomic and mechanical impurities, viscosity, and distribution of the molecular weights of the feed components.^9^ Traditionally, decanted heavy gasoil of catalytic cracking, heavy pyrolysis resin, various highly fragranced low-sulfur petroleum,^10^ and coal pitches^11^ are used to produce needle coke.

Analysis of the results presented in previous works^12−14^ provides insight that, in the future, the replacement of most hydrocarbon resources with alternatives is impossible, so feedstock resources will be relevant for their use in processing, including in the production of petroleum needle coke.

To improve the quality of the needle coke produced and to intensify the development of mesophase, additives of different origins, which have a differentiated action mechanism, are used to improve the quality of the needle coke.

Inorganic additives are of great interest among researchers, and as a rule, they are represented by oxides or other compounds of transition metals, in some cases individual components. In other words, additives of this kind are catalysts that ensure the course of certain reactions under conditions of carbonation. The lack of such additives is to increase the ash of coke and in the requirement of further regenerating additives from the received mesophase pitch, which in the future will be used as a feed for the production of needle coke. A significant effect has been achieved when used as a catalyst for the development of mesophase nickel oxide and cobalt oxide,^15^ which due to their properties are able to accelerate the reactions of polymerization and polycondensation. As a result of these reactions, the resulting mesophase C/H ratio increases, which indirectly indicates the emergence of more condensed structures and a reduction in the proportion of long alkyl chains. Kumar and Srivastava^16^ have conducted investigations on the Cr and Cu effect on the formation of mesophase at 370 °C. The use of these additives has led to the active development of mesophase as well as an increase in the proportion of naphthene hydrocarbons capable of transporting hydrogen, thereby facilitating the carbonation process and short alkyl chains in the mesophase pitch obtained. However, the regeneration of chromium from the mesophase pitch could not be carried out due to its insolubleness in ethanol, which can affect the quality of the received needle coke from the chrome-containing pitch.

In addition to Ni, Co, Cr, and Cu, scientists have investigated the effects of other metals with Lewis acid properties, such as Al and Fe. The work investigates^17^ the process of carbonation in the presence of AlCl_3_; a positive effect has been achieved, but in the media of heavy petroleum residues, AlCl_3_ quickly inactivates transferring into a complex compound, as shown in the article.^18^ The compound of iron, used as a modifier of the development of mesophase, is a metal–organic compound, ferrocene. The investigations^19^ found its active effect on the growth and development of mesophase when added to petroleum residues as well as to coal resins.^20^

The use of organic additives in the coking of hydrocarbons is usually aimed at improving their quality by increasing the proportion of desirable components that contribute to the formation of the developed mesophase and as a result the needle coke. Refs (21) and (22) describe the beneficial effect of the polystyrene additive to pyrolysis resin and coal pitch. Mixing polymer with these two kinds of feedstock helps to improve the texture of the needle coke produced and reduce the coefficient of thermal expansion. The effect of polymer additives has also been investigated in refs (23) and (24) where the authors have identified an increase in the proportion of anisotropic mesophase when added to polyvinyl chloride and increased development of mesophase in the early stages when polyethyleneterephthalate and polystyrene are added, which provides a longer and more complete carbonation process.

From the analysis of the polymer additives used, it can be observed that the greatest positive effect on the development of mesophase is made by polymers consisting of monomers, including cyclical mesogen components. These polymers form active radicals in case of cracking and are easily assimilated in mesophase.^25^

The purpose of this work was to establish the possibility of improving the quality of petroleum needle coke, derived from decanted heavy gasoil catalytic cracking from the FCC (fluid catalytic creaking) unit, using polystyrene as a polymer mesogen additive of up to 15%.

## 2 Materials and Methods

### 2.1 Materials

In this work, decanted heavy gasoil of catalytic cracking (decantoil) was used as the base feedstock of the coking process, obtained during the processing of a kind of West Siberian petroleum mixture at the FCC plant. Quality indices and the decantoil group hydrocarbon composition are presented in Table 1.

**Table 1:** Quality Indices and the Decantoil Group Hydrocarbon Composition

property	value	method
1. Density, at 15 °C (kg·m^3^)	1046.0	GOST 3900-85
2. Coking value (%)	6.11	GOST 19932-99
3. Sulfur content (wt %)	0.13	GOST 51947-2002
4. Dynamic viscosity at 50 °C (mPa·s)	360.0	GOST 25271-93
5. Ash content (wt %)	0.04	GOST 1461-75
6. Flash point (°C)	161	GOST 6356-75
7. Distillation characteristics (vol %):		GOST 2177-82 (method B)
IBP (°C)	300	
5% boil out at	315	
10% boil out at	345	
50% boil out at	414	
FBP (°C)	524	
yield (vol %)	84	
8. Group hydrocarbon composition		SARA analysis
parafino-naphtenic	16.9	
aromatic, including	78.5	
light	3.6	
middle	2.7	
heavy	72.2	
resins	3.1	
asphaltens	1.5	

As a donor of mesogen components, polystyrene in various concentrations was added to the decanted heavy gasoil of catalytic cracking at the stage of delayed coking with the quality indices that are presented in Table 2.

**Table 2:** Polystyrene Quality Indices According to TS 2214-126-05766801-2003

property	regulatory standard	value	method
1. Melt flow rate at 200 °C on a 5 kg load (g/10 min)	from 6.0 to 9.0	7.8	ASTM D 1238
2. Vicat softening point (°C)	no less than 89.0	99.3	ASTM D 1525
3. Izod impact strength, notched (J·m)	no less than 96.0	111.5	ASTM D 256
4. Polish at an angle of 60°	no less than 70.0	70.0	ASTM D 523
5. Residual styrene (wt %)	no less than 0.05	0.04	TS 2214-126-05766801 p.4.10

### 2.2 Delayed Coking and Subsequent Calcination Method

**Figure 1:**
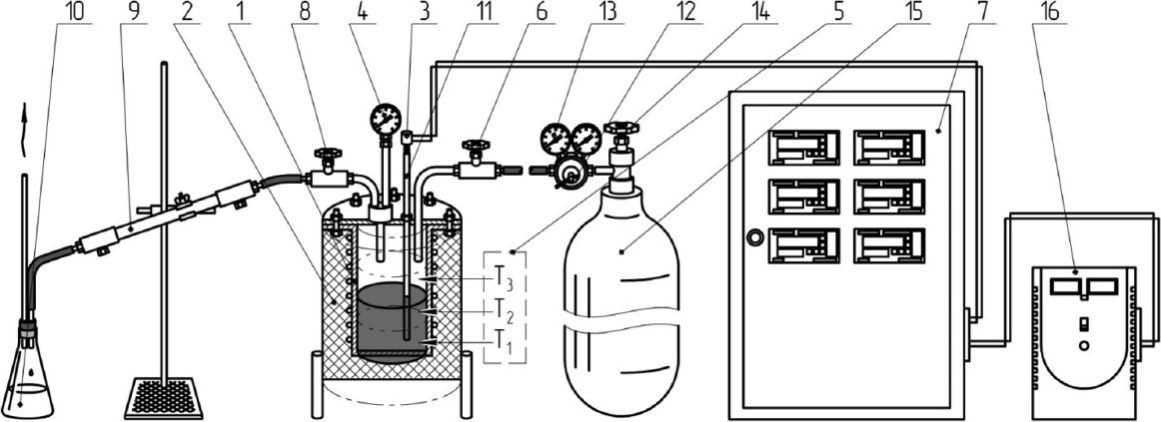
Process flow chart of the laboratory delayed coking unit “UZK-1”: 1, coking reactor; 2, heat insulator; 3, thermocouples of the top and bottom layer of the reactor; 4, rector pressure gauge; 5, three heating zones; 6, pressure test needle valve; 7, electric control unit; 8, reactor needle valve; 9, double-pipe water heat exchanger; 10, distillate receiver; 11, thermocouple pocket; 12, nitrogen reducer (figure drawn up by the authors); 13, pressure test pressure gauge; 14, pressure test valve; 15, nitrogen cylinder for purging and pressure testing; 16, voltage stabilizer.

The delayed coking process was carried out in a laboratory unit, the scheme of which is shown in Figure 1.

Feedstock was loaded into the coking reactor in an amount of 250 g (no more than two-thirds to avoid the coking mass transfer). After that, the top cover was tightly closed using a paranite gasket, and the reactor was pressure-tested with nitrogen. Two thermocouples were placed in a special pocket axially located in the reactor lid so that the first one recorded the temperature of the bottom, and the second one recorded the temperature of the top. The pressure was recorded with a pressure gauge. To carry out heating, the coking reactor was placed in a cylindrical housing equipped with three heating elements. The needle valve for the gas–liquid product mixture outlet, located at the base of the double-pipe water heat exchanger, was used to create a set pressure in the reaction zone. A 0.5 L conical heat-resistant flask was connected in series downstream of the heat exchanger. Gas was released from the distillate receiver using a hose to the exhaust system.

Heating was started after assembling the plant. Decantoil and its mixture coking process with polystyrene were carried out at a coking layer temperature of 495–505 °C and an overpressure of 0.35 MPa. Upon reaching the experimental set pressure, the gas–liquid product mixture formed during coking was gradually vented to the constant pressure value in the reactor.

Upon reaching the experimental set temperature, the system was kept in isothermal mode for 60 min, then the heating was turned off, the pressure was gradually released, and the reactor was cooled down.

Decantoil with the addition of polystyrene in amounts of 0, 2.5, 5.0, 10.0, and 15.0 wt % was used as a feedstock for delayed coking.

Delayed coking process parameters are presented in Table 3.

**Table 3:** Input Parameters of the Delayed Coking Process of Decantoil Mixed with Polystyrene

	composition of raw material						
	decantoil		polystyrene		parameters		
exp	g	wt %	g	wt %	temperature (°C)	overpressure (MPa)	isotherm time (min)
1	250.00	100.0	0.00	0.0	495–505	0.35	60
2	243.75	97.5	6.25	2.5			
3	237.50	95.0	12.50	5.0			
4	225.00	90.0	25.00	10.0			
5	212.50	85.0	37.50	15.0			

The obtained samples of raw (green) needle coke were further subjected to calcination under a nitrogen medium at a temperature of 1250 °C for 60 min. Calcination process parameters are presented in Table 4.

**Table 4:** Calcination Parameters of the Produced Raw Coke Samples

	coke parameters						
			mass loss		calcination parameters		
exp	mass before calcination (g)	mass after calcination (g)	g	%	temperature (°C)	calcination time (min)	
							agency
1	30.03	25.00	5.03	16.75	1250	60	nitrogen
2	35.05	29.96	5.09	14.52			
3	35.00	29.36	5.64	16.11			
4	30.50	26.13	4.37	14.33			
5	30.30	25.77	4.53	14.95			

### 2.3 Method for Microstructure Assessment of Petroleum Needle Coke in Plane-Polarized Light

Microstructure assessment of the obtained needle coke calcined samples was carried out in accordance with GOST 26132-84 “Petroleum and pitch cokes. Microstructure assessment method” using an m-Vizio-MET-222 device. The polished sections (with epoxy resin additives) were made from the material with a fraction of 2–4 mm and a weight of 6–8 g, the obtained polished sections were finished, and the microstructure was assessed using a microvisor in reflected plane-polarized light increased to 90*^x^* to 100*^x^*.

### 2.4 SEM Method for Analyzing Surface Morphology of Petroleum Needle Coke

The structure of petroleum calcined needle decantoil coke was investigated with scanning electron microscopy (SEM) performed by Tescan Vega 3 LMH. An electron microscopic picture of the sample particles was obtained in secondary electrons (SE) in resolution scanning mode, in 16 and 66 μm fields of view. The accelerating voltage was 20 kV, and the emission current was 120 μA. The identification and description of the samples investigated were carried out in accordance with the nomenclature given in a previous work.^26^ The samples were ground in an agate mortar and applied to carbon tape, and the sample was investigated at 40 points. Figures 5–9 show the most typical structure specific to samples of calcined petroleum needle cokes obtained by adding polystyrene of different concentrations to the feedstock.

### 2.5 XRD Method for Analyzing the Fine Structure of the Petroleum Needle Coke

The X-ray structural analysis of petroleum needle coke calcined samples was carried out using an XRD-7000 Shimadzu X-ray powder diffractometer (CuKα radiation, 2.7 kW) at room temperature according to the Debye–Scherrer method. X-ray diffraction patterns were taken with a long accumulation period (2 s) and a scan step of 0.02°. The obtained peaks (reflections) of calcined petroleum needle cokes at maximum on doubled Bragg diffraction angles (2θ) attribute certain structural components.

The obtained reflections of carbon materials at maximum on doubled Bragg diffraction angles (2θ) attribute certain structural components of the samples.

For a detailed assessment of the fine structure of the calcinated petroleum needle cokes by means of the X-ray diffraction method, the interplane distance by diffraction maximum values (002) and (100) and the coherent scattering area in the directions of “*c*” (average crystallite height *L_c_*) and “*a*” (average hexagonal layer diameter *L_a_*) axes were used in this work. To determine the interplane distance (*d*_002_ and *d*_100_) in Å of the obtained raw and calcined samples of petroleum coke, the calculation was carried out according to the Wolf–Bragg equation:^27,28^
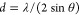
1where λ = 1.5406 is the X-ray wavelength for CuKα (Å) and θ is Bragg’s diffraction angle (rad).

The average linear sizes of crystallites *L_c_* and *L_a_* were determined in Å according to the Scherrer^29^ and Warren equations:^30^
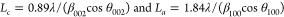
2where 0.89 is the Scherrer constant that is conditionally equal for cokes to ensure uniformity of the published results; 1.84 is the coefficient derived by Warren for the two-dimensional particle size; β is the diffraction line width at the maximum half-height (rad) exclusive of the instrumental peak width *b* = 0.14°.

### 2.6 Dilatometric Investigations of the Linear Thermal Expansion Coefficient of Needle Coke

Determination of the linear thermal expansion coefficient of needle coke samples was performed by means of a Netzsch DIL 402 C dilatometer at a temperature of 40–500 °C and a heating rate of 5 °C·min with blowing air through the furnace space at a flow rate of 50 mL·min. Dilatometry and complex thermal analysis are widely used to describe changes in the physicochemical properties of materials in the processing of carbon and mineral resources.^31−34^

A special tool was used for dilatometric investigation of samples to measure the properties of carbon powders, which is an alundum cylinder with an external diameter of 12 mm and a length of 22 mm; the internal end-to-end channel had a diameter of about 6 mm. The investigated powder was placed in the inner channel and closed on both sides by two alundum pistons with their free ends that extended beyond the linear dimension of the cylinder. This structure presented a mold filled with carbon powder, which could expand freely when heated, and at the same time, it was possible to assess changes in the linear dimensions of the powder sample. A proprietary certified standard alundum sample in the form of a disc 1.01 mm in thickness and 5.00 mm in diameter was used as a reference.

Weighed portions of the investigated powders weighing about 40 mg were placed in a manufactured mold, pre-pressed by a force of about 0.04 kg·mm^2^, and then by means of a micrometer, the thickness of the “measured powders” was assessed at 20 °C with an accuracy of ±0.002 mm. The container with the sample collected in this way was placed in the sample holder of the Netzsch DIL 402 C dilatometer.

Dilatometric tests were carried out in an alundum container with powder thicknesses of 0.98 to 1.03 mm. The sensor for changing the linear dimensions of the sample was pressed by a force of 30 cN. The accuracy of measuring the linear dimensions of the tablet thickness was ±0.125 Nm. For each sample, the values of the linear thermal expansion coefficient were recorded at temperatures of 40, 200, 300, 400, and 500 °C.

### 2.7 XRF Analysis of Sulfur and Microelement Composition of Petroleum Needle Coke

The experimental part on the quantitative determination of sulfur and microelements in the samples of petroleum coke from decantoil was carried out on a sequential wave-dispersive X-ray fluorescence XRF-1800 Shimadzu spectrometer. The instrument was equipped with an X-ray tube with a Rh anode of 3.6 kW.^35,36^ The analysis was carried out without preliminary ashing of the samples using the classical addition method (Ca was added in the form of CaCl_2_). The cathode current was 90 mA, and the tube voltage was 40 kV. The calculations were carried out by the method of fundamental parameters using a standard algorithm for taking into account the effect of the sample carbon matrix on the absorption of X-ray radiation. A weighed portion weighed about 0.5–1 g.

We used a method for calibrating the detector by one element, the essence of which is as follows:

1) CaCl_2_ solution (1 mL) in isopropanol (2 mg of Ca per 1 mL of isoprapanol concentration) was added to the weighed portion of coke and dried at 120 °C.
  Each sample was taken twice. The first time was without an additive, and the second time was with an additive. Moreover, in both cases, the carbon content was postulated to be the same and was taken to be equal to 98 wt %. The remaining 2% were distributed according to the standard algorithm by the method of semi-quantitative analysis between the detected elements.
2) Then, two ratios were calculated: *A* = % Ca/% *S* in the initial sample and *B* = % Ca/% *S* in the sample with the calcium additive. Certainly, *B* is always greater than *A*, for no sulfur was added.
  An equation of the form (*D + A* × *X* × *M*)/(*X* × *M*) *= B* was drawn up, where *D* is the weight of the calcium additive (2 mg), *M* is the sample weight (100 mg), and *X* is the true weight fraction of sulfur in the sample (it is unknown).
3) Solving the equation for *X*, we obtain *X* = *D*/(*M* × (*B* – *A*)) - true weight fraction of sulfur (%): sulfur = 100*X*. The quantitative content of the remaining elements is calculated by the proportion from the data for the sample without an additive. That is, we believe that, if the content of calcium in the analysis results of the sample without an additive is 10 times less than that of sulfur, then its true content is also 10 times less than that of *X*.
4) If the sum of all impurities differs from 2% by more than 10% (that is, more than 2.2 or less than 0.18%), then a correction was made to the carbon content in the sample. Well, that is, if the sum of all the elements equals 3%, then the calculations are repeated from the very beginning with % *C* = 97. The procedure converges. Usually, three to four iterations are required.
5) This method was verified by analyzing artificial carbon mixtures and compared with the classical calibration curve method. It has always appeared to be much better since the concentration ratios are subject to less variation than the concentrations themselves. A similar approach is widely used in quantitative gas chromatographic analysis.

### 2.8 Dynamic Viscosity According to Brookfield

Dynamic viscosity according to Brookfield is designed for testing highly viscous materials, including suspensions, emulsions, and polymers.^37−39^ Determination of dynamic viscosity was performed by means of a Brookfield DV2TLV viscometer using spindles for various viscosity ranges. The feed mixture for determining the viscosity was prepared as follows: polystyrene powder with a particle size of <100 μm was added to the decantoil in the required ratio and, with constant mixing by a magnetic mixer, was heated on a hotplate at a temperature of 200 °C for 120 min to form a homogeneous mixture.

## 3 Results and Discussion

The material balance of coking experiment nos. 1–5 is presented in Table 5. With an increase in the amount of added polystyrene, the release of coking distillates increases from 36.2 to 42.2 wt %, without a significant change in the amount of needle coke formed, due to the involvement of an additional amount of radicals formed during heating and cracking of polystyrene into the system.

**Table 5:** Material Balance of the Decantoil Delayed Coking Mixed with Polystyrene in Various Ratios

	feedstock (wt %)			products (wt %)			
exp	decantoil	polystyrene	input	coke	distillates	gas and losses	output
1	100.0	0.0	100.0	44.8	36.2	19.0	100.0
2	97.5	2.5	100.0	46.0	36.6	17.4	100.0
3	95.0	5.0	100.0	45.6	37.2	17.2	100.0
4	90.0	10.0	100.0	44.4	39.2	16.4	100.0
5	85.0	15.0	100.0	45.2	42.2	12.6	100.0

Quality indices of calcined needle cokes for experiment nos. 1–5 are presented in Table 6. With an increase in the amount of added polystyrene, the actual density of coke increases from 2.0871 to 2.1410 g·cm^3^, which can be explained by the intensification of the coke pitching process during its calcination due to the previously involved mesogen polystyrene components. Quality indices such as the release of volatile substances, ash content, and moisture content comply with the requirements for needle coke for the production of UHP electrodes and further will provide these electrodes with the required operational properties. Since a significant content of volatile substances in the coke material can cause further cracking of the electrode during its operation as a result of the release of volatile components during high-temperature exposure, in turn, a significant ash content negatively affects the conductivity index.

**Table 6:** Quality Indices of Calcined Needle Cokes

		value				
property	method	0.0	2.5	5.0	10.0	15.0
microstructure score	GOST 26132-84	5.4	5.7	6.1	6.2	5.7
CTE from 40 to 500 °C, 10^6^ °C^–1^	ASTM D 6745					
at 40 °C		25,097		24,450	7339	–10,430
at 200 °C		4814		14,006	14,144	11,878
at 300 °C		–19,204		8924	5732	6958
at 400 °C		10,510		12,511	8159	11,320
at 500 °C		4352		5315	5144	9580
absolute density (g·cm^3^)	GOST 10220-82	2.0871	2.1172	2.1293	2.1376	2.1410
volatile-matter content (wt %)	GOST 22898-78	5.27	4.33	4.86	5.14	4.33
ash content (%)	GOST 11022-95	0.05	0.05	0.05	0.04	0.04
moisture (%)	GOST 27589-91	0.05	0.05	0.06	0.06	0.05

**Figure 2:**
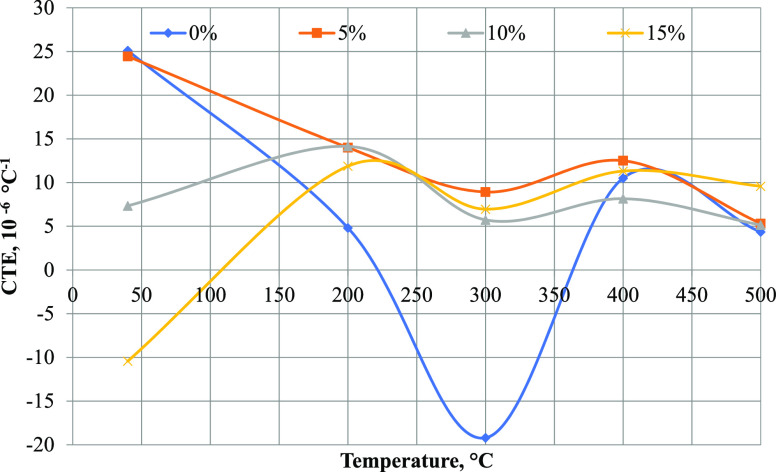
Coefficient of thermal expansion: temperature chart.

**Figure 3:**
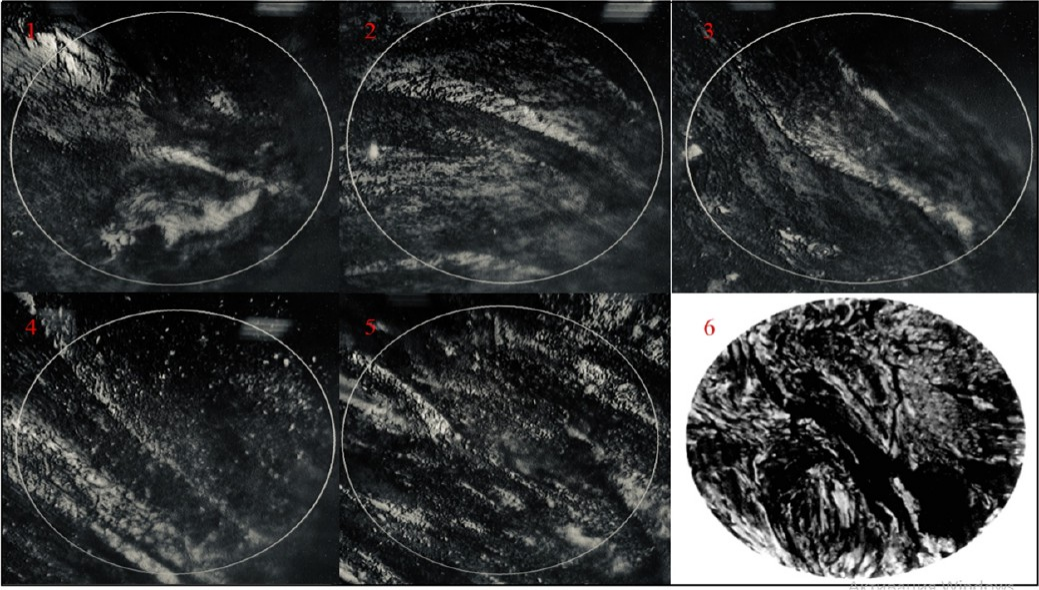
Microstructures of the calcined needle coke samples obtained: 1, exp 1; 2, exp 2; 3, exp 3; 4, exp 4; 5, exp 5; 6, a sample meets six points on the scale of microstructures.

**Figure 4:**
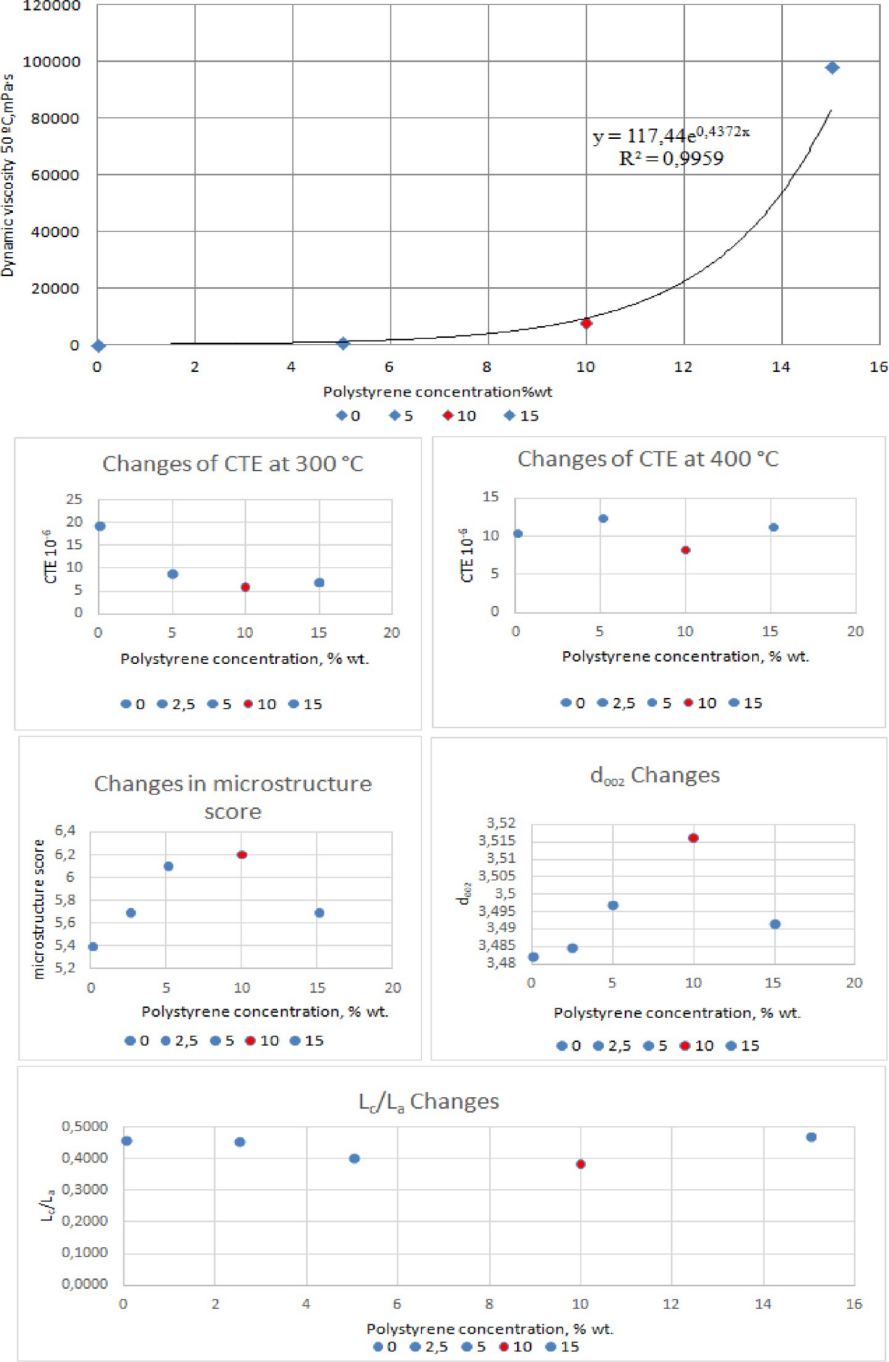
Dynamic viscosity and quality index dependence of calcined cokes on the polystyrene concentration.

The main index that determines the quality of needle coke is the coefficient of thermal expansion; as a result of the tests carried out, there is a tendency for a decrease in the deformation ratio of the carbon material under thermal action with an increase in the proportion of polystyrene in the feedstock. In this case, the minimum CTE 5.732 × 10 ^–6^ °C ^–1^ (at 300 °C) meets a polystyrene concentration of 10 wt % (Figure 2), and a further increase in the polymer content in the feedstock causes an increase in this index. The obtained regularity is kept to a temperature of ∼480 °C. For the calcined cokes obtained in experiment nos. 1–5, the change in the value of the microstructure point also has an extreme approach with an extreme value at a polystyrene concentration of 10 wt %. Thus, the structure of needle coke improves with an increase in the proportion of polystyrene in the feedstock of up to 10 wt % (Figure 3, nos. 1–4), and with a further increase in the polymer concentration (up to 15%), the morphology deterioration is observed (Figure 3, no. 5). The microstructures of needle coke samples are shown in Figure 3. Since the formation of an anisotropic structure requires a long existence of the plastic state of the liquid phase and its low viscosity, then this dependence can be explained by a significant increase in the viscosity of the system due to the addition of a high polystyrene concentration. The limiting stage for liquid-phase thermolysis is diffusion, which is hampered by a significant increase in the viscosity of the system, while a deterioration in the growth and development of the mesophase is observed due to the termination of the coalescence of mesophase spheres and deceleration of these sphere deformations by convective flows. Thus, a highly viscous medium is unfavorable for the formation of a jet structure. To confirm the above, the dynamic viscosity of the mixture at 50 °C of decantoil with various polystyrene concentrations was determined, which concluded that μ_decantoil_ = 130.8 mPa·s, μ_5%ps_ = 1000.0 mPa·s, μ_5%ps_ = 1000.0 mPa·s, μ_10%ps_ = 7350.0 mPa·s, and μ_15%ps_ = 98,200.0 mPa·s. The change in dynamic viscosity depending on the polystyrene concentration in the mixture is shown in Figure 4.

Based on the microelement composition results (Table 7), all the calcined needle coke samples obtained are low-sulfur with a S content of up to 0.5 wt % and meet the requirements for the super-premium coke. In addition, there is an increased sulfur content of 0.31544 wt % in the sample obtained by adding 15 wt % of polystyrene. The obtained peak value of the sulfur content can be explained by the “cellular effect” by limiting the diffusion process in a highly viscous medium with a high concentration of polystyrene. Within the framework of the cellular model, the liquid-phase reaction mechanism is as follows: each of the particles in steps following each other in time *T* randomly moves in the solvent medium. As a result of displacements, random encounters of particles occur, that is, they fall into one cell of the liquid forming a diffusion pair. In a highly viscous medium with polystyrene monomers, the probability of a diffusion pair with sulfur molecules increases, and as a result, more sulfur components remain in the liquid phase and transform into coke.^40^

**Table 7:** Microelement Composition of the Needle Coke Calcined Samples

element	exp 1	exp 2	exp 3	exp 4	exp 5
S (wt %)	0.17876	0.15252	0.14876	0.14427	0.31544
Al (wt %)	0.06569	0.01792	0.01257	0.01163	0.03139
Si (wt %)	0.02748	0.02035	0.01897	0.02089	0.09362
Fe (wt %)	0.01138	0.01966	0.01738	0.01437	0.01842
Ca (wt %)	0.00719	0.00800	0.01143	0.00751	0.01365
K (wt %)	0.00510	0.00346	0.00398	0.00329	0.01409
P (wt %)	0.00259				
Ni (wt %)			0.00164	0.00160	

To confirm the results obtained in the course of examining the microstructure in reflected plane-polarized light, calcined coke samples were analyzed by scanning electron microscopy, and the pictures obtained were interpreted according to the nomenclature presented in a previous work.^26^

**Figure 5:**
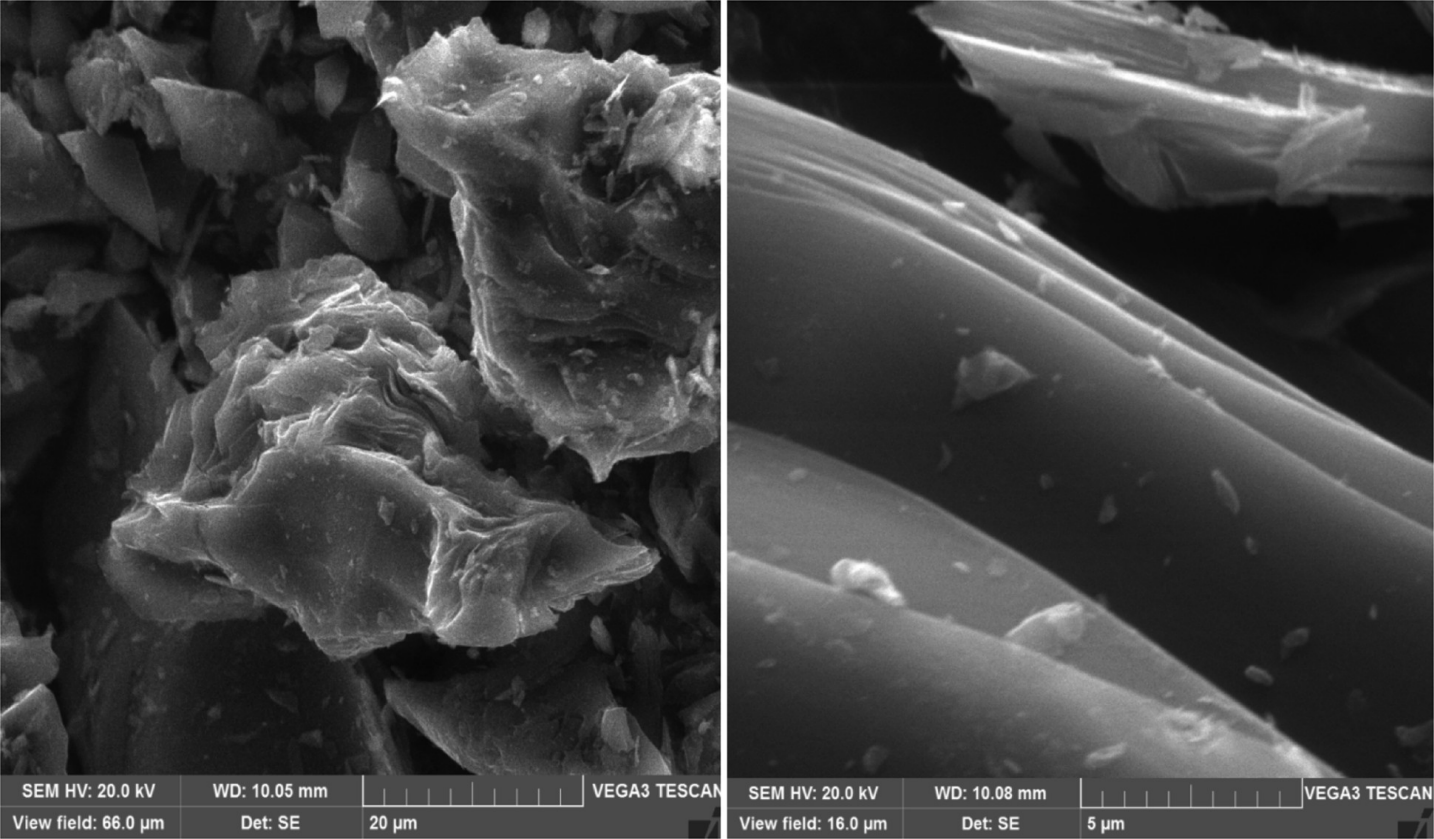
SEM of petroleum needle coke experiment no. 1 (decantoil + 0% PS).

**Figure 6:**
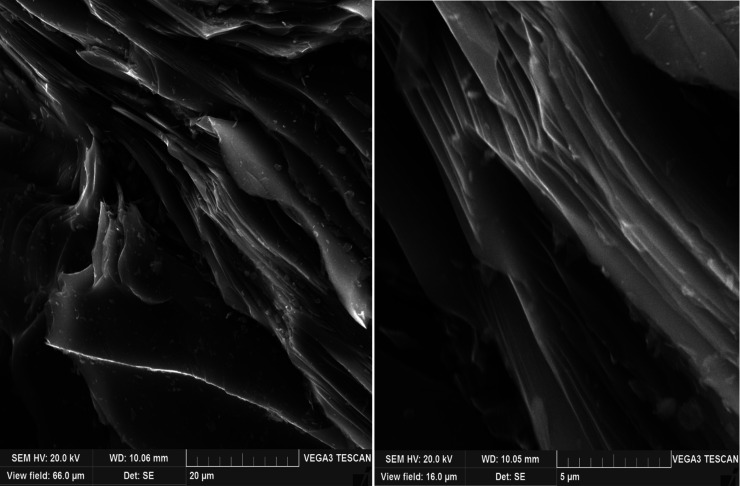
SEM of petroleum needle coke experiment no. 2 (decantoil + 2.5% PS).

**Figure 7:**
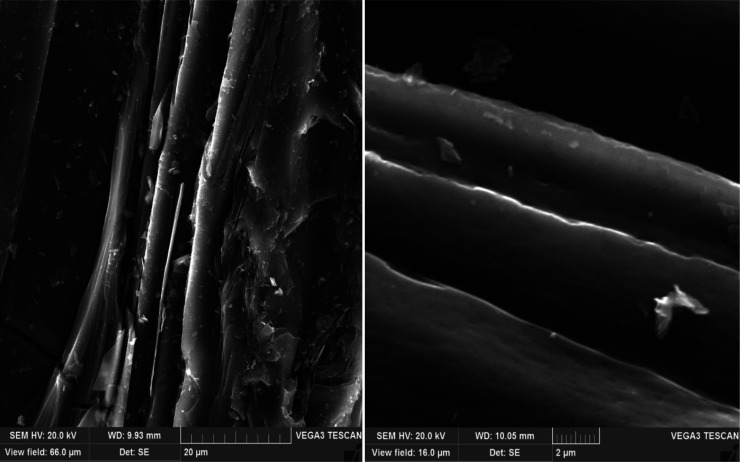
SEM of petroleum needle coke experiment no. 3 (decantoil + 5% PS).

**Figure 8:**
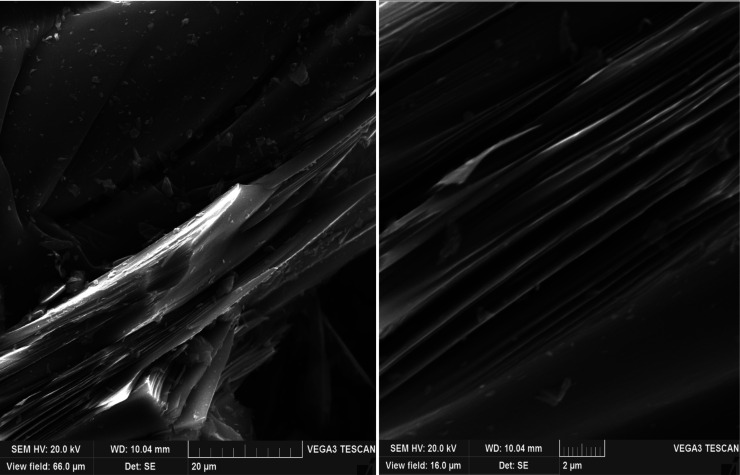
SEM of petroleum needle coke experiment no. 4 (decantoil + 10% PS).

**Figure 9:**
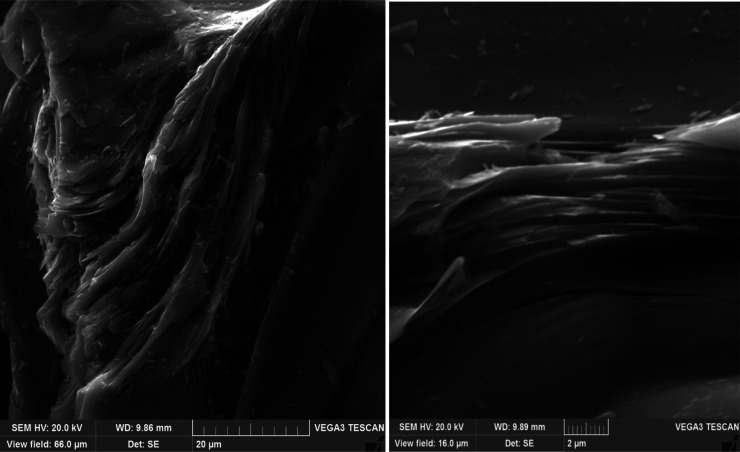
SEM of petroleum needle coke experiment no. 5 (decantoil + 15% PS).

The sample obtained from decantoil without adding polystyrene (experiment no. 1) has an anisotropic regular structure with a predominant anisotropy of the circular flow area (Figure 5). At polystyrene concentrations of 2.5 and 5 wt % (Figures 6 and 7), the structure acquires an anisotropic string approach, while an increase in the polystyrene concentration decreases the coarseness of the fibers and ensures the formation of more elongated lamellae. At a polystyrene concentration of 10 wt %, the structure approaches the super-premium one (Figure 8) with a predominantly smooth string anisotropy of the fibers. When analyzing cokes obtained from a mixture of decantoil with 15 wt % of polystyrene, a coarser fibrous structure with multiple fiber breaks can be observed. Deterioration of the structure with the addition of 15 wt % of polystyrene (Figure 9) is explained by the incomplete development of the mesophase at the stage of liquid-phase thermolysis (due to high viscosity) as well as the possible active evolution of gases at the stage of solidification of the plastic weight due to the high concentration of polystyrene in the system.

For a more complete examination of the fine structure for the calcined samples of needle coke obtained in experiments 1–5 by the X-ray diffraction method, the interplane distances *d*_002_ and *d*_100_ were determined by the value of the diffraction maximum (002) and (100) and crystallite sizes along the “*a*” and “*c*” axes, as presented in Table 8. The obtained linear dimensions and interplane distances comply with the typical samples of needle coke. Based on the results obtained, a slight increase in the interplane distance *d*_002_ from 3.4822 to 3.5161 Å can be observed with an increase in the proportion of polystyrene in the feedstock at a maximum of 10 wt %. The obtained diffraction pattern shows a symmetric form of the 002 peak, which indicates the formation of a structure approaching the graphite one.

**Table 8:** Results of Diffractometric Analysis of Calcined Needle Coke Samples

	reflex (002)				reflex (100)				
exp	2θ (°)	β (°)	*d*_002_ (Å)	*L_c_* (Å)	2θ (°)	β (°)	*d*_100_ (Å)	*L_a_* (Å)	*L_c_*/*L_a_*
1	25.5600	2.2400	3.4822	36.0329	43.0400	2.2300	2.0999	78.4449	0.4593
2	25.5400	2.2300	3.4849	36.1937	42.8800	2.2100	2.1074	77.3571	0.4575
3	25.4500	2.4200	3.4970	33.3361	42.6900	2.1100	2.1163	82.8261	0.4025
4	25.3100	2.3700	3.5161	34.0325	42.7600	1.9700	2.1130	88.7623	0.3834
5	25.4900	2.2000	3.4916	36.6856	42.9400	2.2400	2.1046	78.0665	0.4699

**Figure 10:**
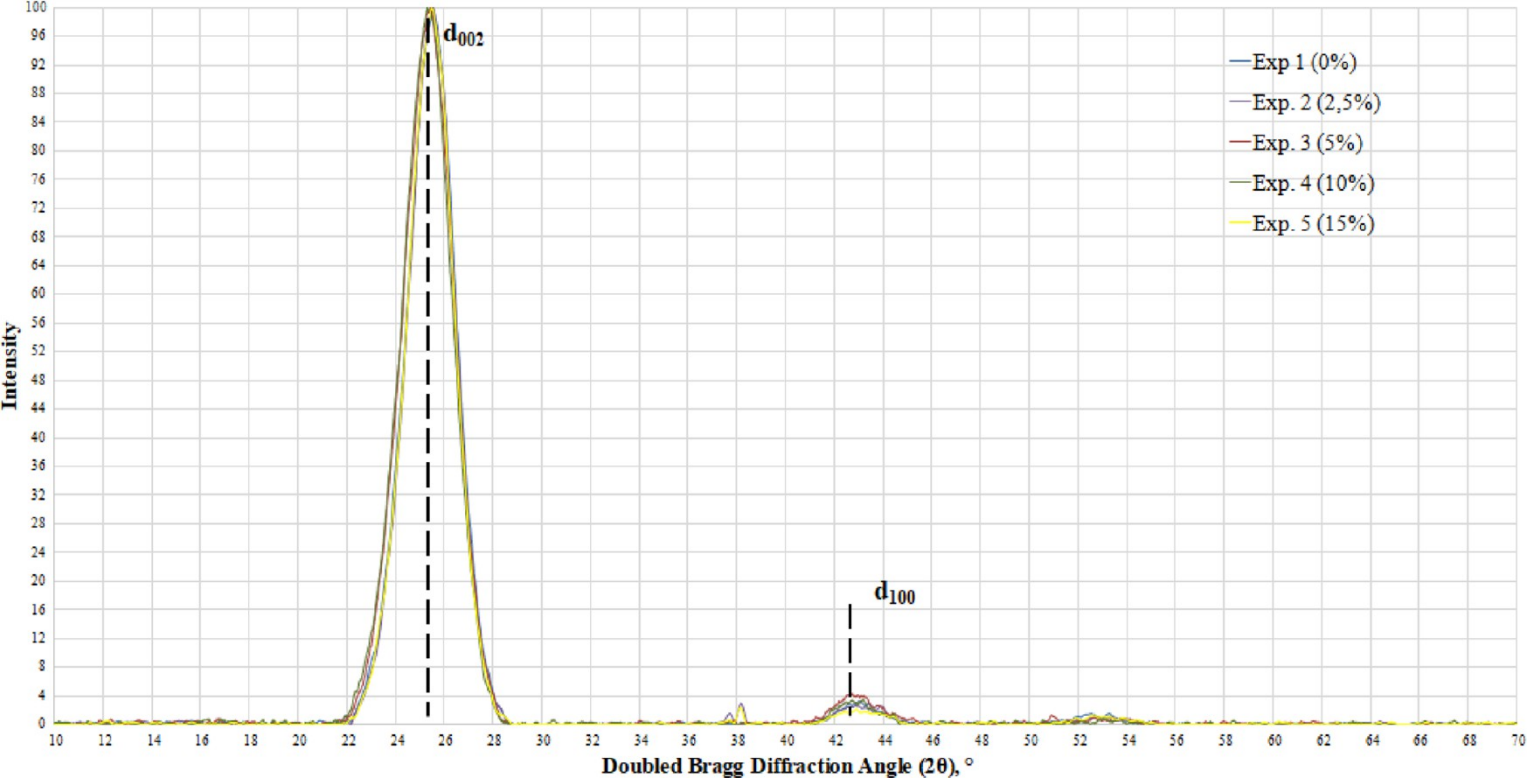
Diffraction patterns of the calcined petroleum needle coke samples obtained.

According to the X-ray diffraction analysis results, the microstructure of the petroleum cokes can be judged on the basis of the ratio of the average height *L_c_* and average diameter *L_a_* of crystallites.^41,42^ Thus, the farther the value of this ratio is from “1”, the more elongated the structure of the fibers and the closer the structure is to the needle coke. With an increase in the polystyrene concentration from 0 to 10 wt %, the ratio of *L_c_* to *L_a_* decreases, moving away from “1” and from 0.4593 to 0.3834, and at 15%, it significantly increases to 0.4699 (Figure 10).

## 4 Conclusions

In the course of the investigation of the effect of the polystyrene additive to the decanted heavy gasoil of catalytic cracking from a mixture of a kind of West Siberian petroleum, a petroleum needle coke was obtained with an improved anisotropic structure and a microstructure point of 6.2 corresponding to the super-premium grades of needle coke. The data obtained as a result of examining the microstructure of coke are confirmed by scanning electron microscopy and X-ray structural analysis. In this case, an increase in the ordering degree of the structure is observed with an increase in the polystyrene concentration of up to 10 wt %, and with further addition of the polymer, the ordering degree of the coke decreases due to a significant change in the viscosity parameters of the system. Such an extreme dependence is observed in the X-ray diffraction analysis of cokes (the ratio of the linear dimensions of the crystallite and the interplane distances), scanning electron microscopy (the transition from the circular area anisotropy to the string one), and examining of the coke microstructure in reflected plane-polarized light. A similar dependence is the coefficient of thermal expansion of the obtained needle coke samples in the temperature range of 250–480 °C, while the lowest CTE for the sample obtained with the addition of 10 wt % polystyrene at 300 °C is 5.732 × 10^–6^ °C^–1^. Thus, polystyrene acts as a modifying agent, which obviously provides the involvement of additional active fragranced radicals into the system, which provide the starting development of the mesophase at earlier stages, thereby increasing the time interval for the development of plastic weight and increasing the petroleum needle coke quality to a super-premium one.
